# Lower serum cholesterol levels as a risk factor for critical illness polyneuropathy: a matched case–control study

**DOI:** 10.1038/s41598-023-47232-3

**Published:** 2023-11-21

**Authors:** Gudrun Zulehner, Stefan Seidel, Alexander Polanz, Christian Schörgenhofer, Paulus Rommer, Marieke Merrelaar, Dominik Roth, Harald Herkner, Sybille Behrens, Calvin Lukas Kienbacher

**Affiliations:** 1 Department of Neurology, Medical University of Vienna, Waehringer Guertel 18-20, 1090 Vienna, Austria; 2 Department of Clinical Pharmacology, Medical University of Vienna, Waehringer Guertel 18-20, 1090 Vienna, Austria; 3 Department of Emergency Medicine, Medical University of Vienna, Waehringer Guertel 18-20, 1090 Vienna, Austria

**Keywords:** Diseases, Health care, Medical research, Neurology, Pathogenesis, Risk factors

## Abstract

Critical illness polyneuropathy (CIP) is a frequent and underdiagnosed phenomenon among intensive care unit patients. The lipophilic nature of neuronal synapses may result in the association of low serum cholesterol levels with a higher rate of CIP development. We aimed to investigate this issue in critically ill patients. All cases diagnosed with CIP in our tertiary care hospital between 2013 and 2017 were 1:1 matched with controls without the condition by age, sex, and ICD diagnoses. The main risk factors examined were the differences in change between initial and minimum serum total cholesterol levels, and minimum serum total cholesterol levels between matched pairs. Other predictors were serum markers of acute inflammation. We included 67 cases and 67 controls (134 critically ill patients, 49% female, 46% medical). Serum total cholesterol levels decreased more profoundly in cases than controls (median: −74 (IQR −115 to −24) vs. −39 (IQR −82 to −4), median difference: −28, 95% CI [−51, −5]), mg/dl). Minimum serum total cholesterol levels were lower in the cases (median difference: −24, 95% CI [−39, −9], mg/dl). We found significant median differences across matched pairs in maximum serum C-reactive protein (8.9, 95% CI [4.6, 13.2], mg/dl), minimum albumin (−4.2, 95% CI [−6.7, −1.7], g/l), decrease in albumin (−3.9, 95% CI [−7.6, −0.2], g/l), and lowest cholinesterase levels (−0.72, 95% CI [−1.05, −0.39], U/l). Subsequently, more pronounced decreases in serum total cholesterol levels and lower minimum total cholesterol levels during critical care unit hospitalizations may be a risk factor for CIP.

## Introduction

Intensive care unit acquired weakness (ICUAW), including critical illness polyneuropathy (CIP), is a condition of significant burden, affecting up to 100% of critical care unit patients, depending on the underlying condition^1–3^. It increases short-term mortality and the risk of long-term disability^4^. Furthermore, difficulties in weaning from the ventilator occur frequently^5^. Medical history and a clinical evaluation are the diagnostic cornerstones^6^. The hallmark of CIP is a symmetric, predominantly proximal weakness of all limbs. In contrast, ocular muscles are usually spared. The neurologic examination might show lost or reduced tendon reflexes^1^. Noteworthy, critical illness polyneuropathy and myopathy might be difficult to differentiate and may occur concomitantly. In contrast to critical illness myopathy, sensory changes such as numbness, tingling sensations, and pain might be present in CIP ^2^. Beside the clinical aspects, nerve conduction studies (NCS) are helpful in diagnosing this condition, affection of sensory nerves supports the discrimination from isolated myopathy^4^. Nerve conduction studies reveal axonal alterations as the hallmark of CIP, whereas demyelination suggests a different etiology of neuropathy^7,8^. NCS require specialized equipment and well-trained personnel. These resources might be sparse in many places worldwide. In addition, several factors may substantially interfere with the quality and interpretation of NCS results. These include edema, which is frequently seen in critical care unit (CCU) patients.

The patient’s age and comorbidities are important risk factors for CIP^1,9^. Furthermore, prior studies have shown that especially critically ill individuals with inflammatory processes, such as sepsis, are prone to develop CIP^10^. Commonly used inflammation parameters to screen for infections include C-reactive protein (CRP), albumin, cholinesterase, fibrinogen levels, and leukocyte counts^11–13^.

Potential therapeutic regimens to treat or reduce the risk of developing CIP include adapted nutrition, antioxidants, insulin substitution, and immunoglobulins^14,15^. However, the effects of these interventions are limited. Physiotherapy, the renunciation of relaxation, and minimizing ventilator time remain the most critical prophylactic approaches.

Cholesterol is widely distributed in cell membranes, including neurons and myelin sheaths^16^. More than a quarter of the total body cholesterol is located in the brain and peripheral nerve cells^17^. Besides protecting the structural integrity of cells, lipids fulfill various other functions in synaptic interactions, signal transmission, and membrane trafficking^18^. Data from patients with type 2 diabetes indicate that low serum cholesterol levels may be associated with progression of diabetic polyneuropathy^19^. The FDA has also warned about the potentially harmful effects of hypocholesterinemia resulting from the use of Proprotein convertase subtilisin-kexin type 9 (PCSK9) inhibitors and statins in 2012 and 2014^20,21^. Contrarily, two recent reviews did not find any detrimental neurological consequences of treatment with statins and even proved a beneficial effect on cognitive functions^22,23^.

Although the neurological safety profile of long-term antilipidemic pharmacotherapy has been investigated, their findings might not be extrapolatable to hypocholesterolemia in intensive care unit patients. Generalized inflammation and the deficiency of vitally important cholesterol during critical illness might be risk factors for CIP. To our knowledge, no study has yet investigated the association between hypocholesterolemia and this condition.

## Methods

In our case–control study at an academic, high-volume, tertiary healthcare facility, we included all adult (above 18 years of age) CCU patients with a diagnosis of CIP between 2013 and 2017 in our retrospective analysis. The prerequisite for the cases to be included in the study was that the patients had an NCS measurement on at least one sensory and motor nerve on their upper and lower extremity performed during their stay. Cases were defined as patients who were diagnosed with CIP based on NCS and clinical assessment by a neurologist according to standard diagnostic criteria^6^. All included cases showed clinical signs of CIP, e.g., weakness and/or difficulties in weaning from the ventilator. CCU patients without signs of CIP served as potential controls, there was no clinical suspicion of ICUAW in these patients. We carefully screened the electronic records, including the physiotherapists’ documentation of all controls for any clinical evidence of CIP. Cases were matched to controls in a 1:1 ratio using the best-match principle regarding sex, International Statistical Classification of Diseases and Related Health Problems (ICD) diagnoses^24^, and age. Regarding diagnoses, we chose the maximum number of ICD subdomains, described by the initial number or letter of the code (i.e., 9, A to N, Q to T, and Z), in common between cases and potential controls as the qualifier for matching. Every control could only serve as a matching partner for a single case. Data was extracted from digital patient records.

We compared cases and controls in regard of change of serum total cholesterol levels using quantile regression. Furthermore, accounting for our matched design, we used conditional logistic regression analyses to estimate the effect of cholesterol in CIP. We used the outcome CIP (case vs. control) as the dependent variable, and change in serum cholesterol levels, whether the patient took lipid-lowering medication at admission or received lipid-containing parenteral nutrition during hospitalization, as well as the length of CCU stay as independent variables. We chose the decrease from initial to minimum total cholesterol levels as the main risk factor.

We also investigated minimum levels of total serum cholesterol during the stay at the hospital as a predictor. Furthermore, we examined the following serum markers of acute inflammation: Albumin, cholinesterase, fibrinogen, CRP concentrations, and leukocyte counts. Owing to the non-normal distribution of the majority of continuous data, we present the median with the 25% to 75% interquartile range for sample data. To estimate the difference across case–control pairs, we again used quantile (median) regression with robust 95% confidence intervals (95% CI) of the median.

We used a last observation carried forward approach for missing values and conducted a sensitivity analysis to assess the robustness of our approach.

We did not perform a formal sample size calculation, because (1) the effect of cholesterol on the incidence of CIPNP has not been described yet, and (2) because the reported incidence of CIPNP has an extreme range from 25 to 100 per 100 patients^3,25^. Therefore, we decided to include all available cases from our institution, which we expected to equal approximately n = 60, with a reasonable chance to find matching controls in a 1:1 ratio. As our analyses were based on regression models, we used the rule of thumb that 10 cases per co-variable should yield sufficient power in a multivariable model, which would have allowed for 6 co-variables within one model.

We used MS Excel (Microsoft Corporation) and Stata 17MP (Stata Corporation) for data management and analysis. The STROBE statement for our manuscript can be found in Supplementary Table S1.

We conducted this study in compliance with the principles of the Declaration of Helsinki. The study’s protocol was reviewed and approved by the ethics committee of the Medical University of Vienna. Informed consent was waived by the abovementioned ethics committee of the Medical University of Vienna (EK 2042/2017).

## Results

CIP was diagnosed in a total of 89 patients. Of those, 22 individuals were excluded from the study due to a lack of matching controls. Ultimately, we included 134 patients (67 cases, 67 controls, 66 (49%) female, 73 (54%) surgical, 61 (46%) medical) into our analysis. Table 1 provides the baseline characteristics of the study population.

**Table 1:** Baseline characteristics of the study population.

Characteristics	Overall Population (N = 134)	CIP Cases (n = 67)	Controls (n = 67)
Female, n (%)	66 (49)	33 (49)	33 (49)
Age, years, median (IQR)	63 (52 to 71)	64 (52 to 71)	63 (52 to 71)
surgical CCU patients, n (%)	73 (54)	33 (49)	40 (60)
medical CCU patients, n (%)	61 (46)	34 (51)	27 (40)
Weight, kg, median (IQR)	75 (64 to 87)	79 (65 to 90)	74 (62 to 84)
Height, cm, median (IQR)	168 (160 to 175)	170 (165 to 178)	165 (153 to 175)
Body mass index, kg/m^2^, median (IQR)	25.9 (22.9 to 30.6)	25.4 (22.6 to 31.1)	26.1 (22.9 to 30.3)
Length of CCU stay, days, median (IQR)	25 (8 to 46)	44 (26 to 68)	8 (1 to 22)
Length of hospital stay, days, median (IQR)	68 (32 to 114)	84 (36 to 119)	58 (31 to 114)
Lipid lowering medication at admission, n (%)	27 (20)	16 (24)	11 (16)
Parenteral nutrition with lipid emulsion, n (%)	65 (49)	38 (57)	27 (40)

*CCU* critical care unit, *CIP* critical illness polyneuropathy, *IQR* interquartile range, *Kg/m*^*2*^ kilogram/square meter.

**Figure 1:**
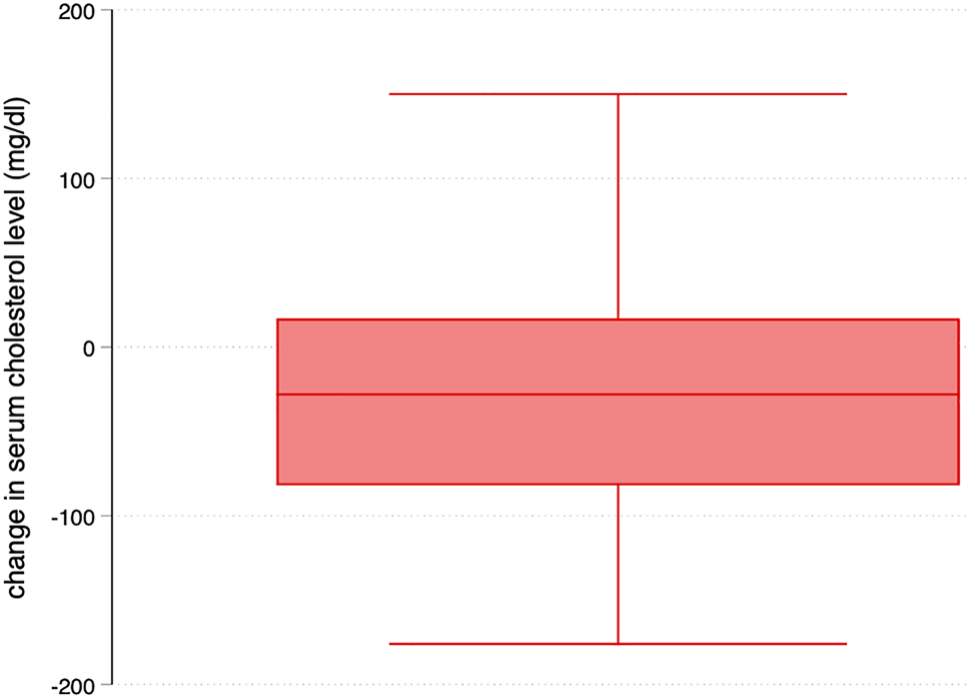
Difference in change in serum total cholesterol level between matched cases and controls. mg/dl, milligram/deciliter.

Median initial serum cholesterol levels were 149 (IQR 110–187) mg/dl in the cases and 146 (IQR 106–170) mg/dl in the controls (median difference across case–control pairs 5, 95% CI [−16, 26], mg/dl). Decreases in serum cholesterol levels were significantly more pronounced in the cases (−74 (IQR −115 to −24) mg/dl) than in the controls (-39 (IQR −82 to −4) mg/dl, median difference across case–control pairs: −28, 95% CI [−51, −5], mg/dl). (See also Fig. 1 and Table 2) Despite higher initial levels of total cholesterol (149 (IQR 110 to 187) vs. 146 (IQR 106 to 170) mg/dl; median difference across case–control pairs: 5, 95% CI [−16, 26], mg/dl), minimum cholesterol levels were significantly lower in the cases (62 (IQR 45 to 86) vs. 88 (IQR 64 to 129) mg/dl in controls; median difference across case–control pairs: −24, 95% CI [−39, −9], mg/dl). The matched difference in change in serum total cholesterol levels between groups is depicted in Fig. 1. The crude decreases in serum total cholesterol levels of cases and controls are presented in Supplementary Figure S1. Details regarding lipid subtypes can be found in Supplementary Table S2.

**Table 2:** Primary predictor.

Predictor	Overall population	CIP cases	Controls	Median matched pair difference cases vs. controls [95% CI]
Initial total cholesterol, mg/dl, median (IQR)	147 (110 to 174)	149 (110 to 187)	146 (106 to 170)	5 [−16, 26]
Minimum total cholesterol, mg/dl, median (IQR)	75 (55 to 112)	62 (45 to 86)	88 (64 to 129)	−24 [−39, −9]**
Decrease in total cholesterol, mg/dl, median (IQR)	−52 (−99 to −15)	−74 (−115 to −24)	−39 (−82 to −4)	−28 [−51, −5]*
Days between initial and minimum total cholesterol, mg/dl, median (IQR)	11 (4 to 28)	10 (4 to10)	11(5 to 32)	−2 [−7, 3]

*CIP* critical illness polyneuropathy, *CI* confidence interval, *IQR* interquartile range, *Mg/dl* milligram/deciliter.

*p = 0.02, **p = 0.002.

A conditional logistic regression model controlling for lipid lowering medication, lipid emulsion nutrition, and the patients’ length of CCU stay showed a 1% increase in odds (odds ratio 1.01, 95% CI [0.998, 1.022]) for the development of CIP for every 1 mg/dl decrease in serum total cholesterol levels between the initial and lowest concentrations. This effect did not considerably change after adjusting for missing values (odds ratio 1.016, 95% CI [0.992, 1.04]). However, the respective results were not significant.

The maximum C-reactive protein levels (median difference across case–control pairs 8.9, 95% CI [4.56, 13.24], mg/dl), the decreases in albumin (median difference across case–control pairs: −3.9, 95% CI [−7.6, −0.2], g/l), as well as the minimum levels of albumin (median difference across case–control pairs: −4.2, 95% CI [−6.7, −1.7], g/l) and cholinesterase (median difference across case–control pairs: −0.72, 95% CI [−1.05, −0.39 U/l], U/l) differed significantly between the matched pairs. Minimum leukocyte counts (across case–control pairs: −0.75, 95% CI [−1.77, 0.27], G/l) and fibrinogen levels (across case–control pairs: 28, 95% CI [−28, 84], mg/dl), as well as the decreases in leukocyte count (across case–control pairs: −1.68, 95% CI [−4.49, 1.13], G/l) and fibrinogen (across case–control pairs: −19, 95% CI [−99, 61], mg/dl) differed insignificantly between the matched patients. Table 3 displays the secondary findings in detail.

**Table 3:** Secondary predictors.

Predictor	Overall population	CIP cases	Controls	Median matched pair difference cases vs. controls [95% CI]
Initial C-reactive protein, mg/dl, median (IQR)	1.5 (0.5 to 7.9)	1.6 (0.6 to 9.6)	1.2 (0.3 to 6.5)	0.05 [−1.9, 2.0]
Maximum C-reactive protein, mg/dl, median (IQR)	25.1 (17.5 to 30.9)	27.1 (21.3 to 36.0)	22.0 (15.8 to 27.3)	8.9 [4.6, 13.2]*
Initial albumin, g/l, median (IQR)	36.4 (30.2 to 41.2)	36.3 (29.3 to 40.2)	36.5 (31 to 42.1)	−1.6 [−4.3, 1.1]
Minimum albumin, g/l, median (IQR)	19.9 (16.9 to 23.9)	18.6 (15.6 to 20.9)	22.5 (17.9 to 25.4)	−4.2 [−6.7, −1.7]*
decrease in albumin, g/l, median (IQR)	−16.4 (−20.2 to −10)	−17.2 (−21.8 to −9.6)	−14.9 (−19.4 to −10)	−3.9 [−7.6, −0.2]**
Initial cholinesterase, U/l, median (IQR)	4.4 (3.1 to 6.3)	4.6 (2.9 to 6.6)	5.19 (3.6 to 6.8)	−0.1 [−1.0, 0.8]
Minimum cholinesterase, U/l, median (IQR)	1.9 (1.2 to 2.8)	1.5 (1 to 2.3)	2.4 (1.6 to 3.4)	−0.7 [−1.15, −0.4]*
Decrease in cholinesterase, U/l, median (IQR)	−2.7 (−4.4 to −1.2)	−2.7 (−4.9 to −1.2)	−2.5 (−4.1 to −0.9)	−0.6 [−1.6, 0.4]
Initial leukocyte count, G/l, median (IQR)	10.2 (7.1 to 14.2)	10.4 (7.4 to 14.8)	9.7 (7.0 to 13.9)	2.2 [−0.1, 4.3]
Minimum leukocyte count, G/l, median (IQR)	4.7 (2.9 to 5.9)	3.9 (2.3 to 6.2)	4.9 (3.3 to 5.8)	−0.75 [−1.8, 0.3]
Decrease in leukocyte count, G/l, median (IQR)	−5.0 (−9.7 to −2.8)	−5.7 (−10.7 to −3.2)	−4.4 (−8.6 to −1.9)	−1.7 [−4.5, 1.1]
Initial fibrinogen, mg/dl, median (IQR)	426 (333 to 559)	449 (350 to 590)	401 (309 to 559)	40 [−56, 136]
Minimum fibrinogen, mg/dl, median (IQR)	246 (181 to 299)	251 (184 to 307)	233 (175 to 294)	28 [−28, 84]
Decrease in fibrinogen, mg/dl, median (IQR)	−176 (−306 to −60)	−205 (−310 to −82)	−151 (−306 to −28)	−19 [−99, 61]

*CI* confidence interval, *CIP* critical illness polyneuropathy, *G/l* giga (1000)/liter, *g/l* gram/liter, *IQR* interquartile range, *Mg/dl* milligram/decilitre, *U/l* units/liter.

*p ≤ 0.001, **p < 0.05.

The median length of stay at a critical care unit was 44 (IQR 26 to 68) days in the CIP group, and therewith significantly longer compared to 8 (IQR 1 to 22) days in the control group (median difference across case–control pairs: 30, 95% CI [23, 38], days). The median length of hospital stay was also longer in the CIP group: 84 (IQR 36 to 119) vs. 58 (31 to 114) days (median difference across case–control pairs: 9, 95% CI [−7, 24], days).

## Discussion

We found significant differences in the decreases in serum total cholesterol levels between intensive care unit patients with and without CIP. This leads to the assumption that fast and severe drops in serum lipid levels might be a major risk factor for CIP development.

Our hospital houses over 130 CCU beds with an annual turnover of more than 2,000 patients. Nevertheless, only 98 cases of CIP were recorded in our database of nerve conduction studies over the five-year period. This number reflects substantially fewer individuals than expected when reviewing the literature^2^. One reason is that nerve conduction studies are not performed in every patient with clinical signs of weakness, including CIP, in our setting. These patients didn’t meet our inclusion criteria.

We are aware of the fact that our study has some limitations. One might argue that the cases in our study were sicker and therefore more likely to develop CIP than the controls, as indicated by their longer duration of stay at CCUs. On the other hand, to our knowledge, the underlying pathophysiological mechanisms of the development of CIP during this longer stay are not fully understood. Nutrition and therefore also the lipid status plays a pivotal role in critically ill patients. A recent study in a real-world cohort demonstrated that early enteral nutrition is associated with improved outcomes in critically ill patients^26^, emphasizing the importance of this topic.

Furthermore, a prospective study showed early alterations in NCS measurement in critically ill patients as early as within a few days after admission^10^. These findings were also associated with higher mortality, which demonstrates the rapid effects of critical illness on nerve cells in the early phase of CCU treatment. However, the clinical discrimination between CIP and generalized weakness due to the underlying condition might be challenging.

Due to our study’s retrospective design, we cannot rule out that patients included as controls developed CIP after hospital discharge or had minor forms of the condition. However, we believe the latter to be a minor issue as we thoroughly screened all patient records for evidence of CIP.

Another important aspect is that the pathogenesis of CIP is likely multifactorial. Severe drops in serum lipid levels can be caused by critical illness itself^27^. This might lead to a breakdown of nerve cells or impair the communication between them, e.g., by generalized inflammation and capillary leak^1,28^. This could possibly explain why the cases had a longer stay at the CCU, lower cholesterol levels and were more prone to develop CIP subsequently. Due to the lack of data in our retrospective analysis, we cannot safely rule out other potentially contributing factors, e.g., fasting blood glucose or vitamin B_12_, although all available levels of the latter (23% of our study population) were in the normal range.

Our results are consistent with a prior study, indicating that hypocholesterinemia might result in aggravated polyneuropathy^19^. This trial was conducted in non-critically ill type 2 diabetes patients. Nevertheless, the mechanism discussed above might be similar in our study population: The lipid composition of Schwann cells might be altered by cholesterol deficiency^28^.

Contrarily, a recent review of animal studies reports that cholesterol depletion and synthesis inhibition may enhance axonal regeneration^29^. Furthermore, cholesterol synthesis was reduced after neural injury in a rat model^30^. It remains unclear whether these findings are also applicable to humans.

Finally, the duration of critical illness is a significant risk factor for CIP^4^. To summarize, our findings from a dataset of multidisciplinary intensive care unit patients suggest that low serum cholesterol levels and profound decreases of cholesterol levels are associated with critical illness development. Future prospective trials are needed to elaborate if there is a causal relationship between these entities.

### Supplementary Information

Supplementary Information.

## Data Availability

The datasets generated and/or analysed in this study are available from the corresponding author on reasonable request.
